# Methylation in *Mycobacterium tuberculosis* is lineage specific with associated mutations present globally

**DOI:** 10.1038/s41598-017-18188-y

**Published:** 2018-01-09

**Authors:** Jody Phelan, Paola Florez de Sessions, Leopold Tientcheu, Joao Perdigao, Diana Machado, Rumina Hasan, Zahra Hasan, Indra L. Bergval, Richard Anthony, Ruth McNerney, Martin Antonio, Isabel Portugal, Miguel Viveiros, Susana Campino, Martin L. Hibberd, Taane G. Clark

**Affiliations:** 1 Faculty of Infectious and Tropical Diseases, London School of Hygiene and Tropical Medicine, London, United Kingdom; 2 Genome Institute of Singapore, Biopolis, Singapore; 3 Vaccines and Immunity Theme, Medical Research Council Unit, Fajara, The Gambia; 4 iMed.ULisboa - Research Institute for Medicines, Faculdade de Farmácia, Universidade de Lisboa, Lisboa, Portugal; 5 Unidade de Microbiologia Médica, Global Health and Tropical Medicine, Instituto de Higiene e Medicina Tropical, Universidade Nova de Lisboa, Lisboa, Portugal; 6 Department of Pathology and Laboratory Medicine, The Aga Khan University, Karachi, Pakistan; 7 Royal Tropical Institute, KIT Biomedical Research, Amsterdam, The Netherlands; 8 Lung Infection and Immunity Unit, UCT Lung Institute, University of Cape Town, Cape Town, South Africa; 9 Faculty of Epidemiology and Population Health, London School of Hygiene and Tropical Medicine, London, United Kingdom

## Abstract

DNA methylation is an epigenetic modification of the genome involved in regulating crucial cellular processes, including transcription and chromosome stability. Advances in PacBio sequencing technologies can be used to robustly reveal methylation sites. The methylome of the *Mycobacterium tuberculosis* complex is poorly understood but may be involved in virulence, hypoxic survival and the emergence of drug resistance. In the most extensive study to date, we characterise the methylome across the 4 major lineages of *M*. *tuberculosis* and 2 lineages of *M*. *africanum*, the leading causes of tuberculosis disease in humans. We reveal lineage-specific methylated motifs and strain-specific mutations that are abundant globally and likely to explain loss of function in the respective methyltransferases. Our work provides a set of sixteen new complete reference genomes for the *Mycobacterium tuberculosis* complex, including complete lineage 5 genomes. Insights into lineage-specific methylomes will further elucidate underlying biological mechanisms and other important phenotypes of the epi-genome.

## Introduction

Tuberculosis disease (TB) caused by pathogens of the *Mycobacterium tuberculosis* complex (MTBC) are an important global public health issue worldwide, with >9 million new cases and 1.7 million deaths each year^1^. A combination of the increasing prevalence of anti-tuberculosis drug resistance, HIV/AIDS infection interaction, and an under-equipped arsenal - requiring new effective treatments and vaccines, are a major barrier to disease control. The *M*. *tuberculosis* genome (size 4.4 Mb, GC-content 60%) is characterised by low sequence diversity^2^, with known variation between stain-types, including between three ‘ancient’ (1, 5, 6), three ‘modern’ (2, 3, 4), and one intermediate lineage (7)^3^. The lineages vary in propensity to transmit and cause disease^4^; with modern strain lineages, including Beijing strains, being more successful in terms of their geographical spread and have a shorter latency in humans^5^. However, results are inconsistent and there is considerable inter-strain variation within lineages, which is difficult to explain in the context of the low sequence diversity^6^.

Several lines of evidence have revealed N6-methyladenine (^m6^A) and 5-methylcytosine (^m5^C) methylation mechanisms within *M*. *tuberculosis* genomes. Motifs within three DNA methyltransferases (MTases), *mamA*, *mamB*, *hdsS*.*1*, *hsdM*, and *hsdS* are responsible for m6A modification^7,8^. *MamA* also influences gene expression in *M*. *tuberculosis* and plays an important but strain-specific role in fitness during hypoxia, promoting survival in discrete host microenvironments^7^. A recent study examining the methylation patterns in 12 diverse members of the MTBC using PacBio technology reported the presence of three unique methylation motifs and associated these to their respective MTases^8^. They found a number of isolates for which methylation of specific motifs were completely absent from the genome. It was suggested that mutations in the MTases cause a loss of function and remove methylation from the genome. Genetic and potentially transcriptomic differences, may play important roles in determining the clinical outcome differences observed between these strains. Genetic differences may be further modified by epigenetic mechanisms, as observed in other bacterial species^9^, however methylome data has rarely been considered for the *M*. *tuberculosis* complex. Here we present to our knowledge the largest and most diverse study of methylation in *M*. *tuberculosis* using PacBio technology, and identify key mutations in associated genes, which appear to be present across a phylogeny based on a global set of isolates.

## Results

### New reference genomes

**Figure 1:**
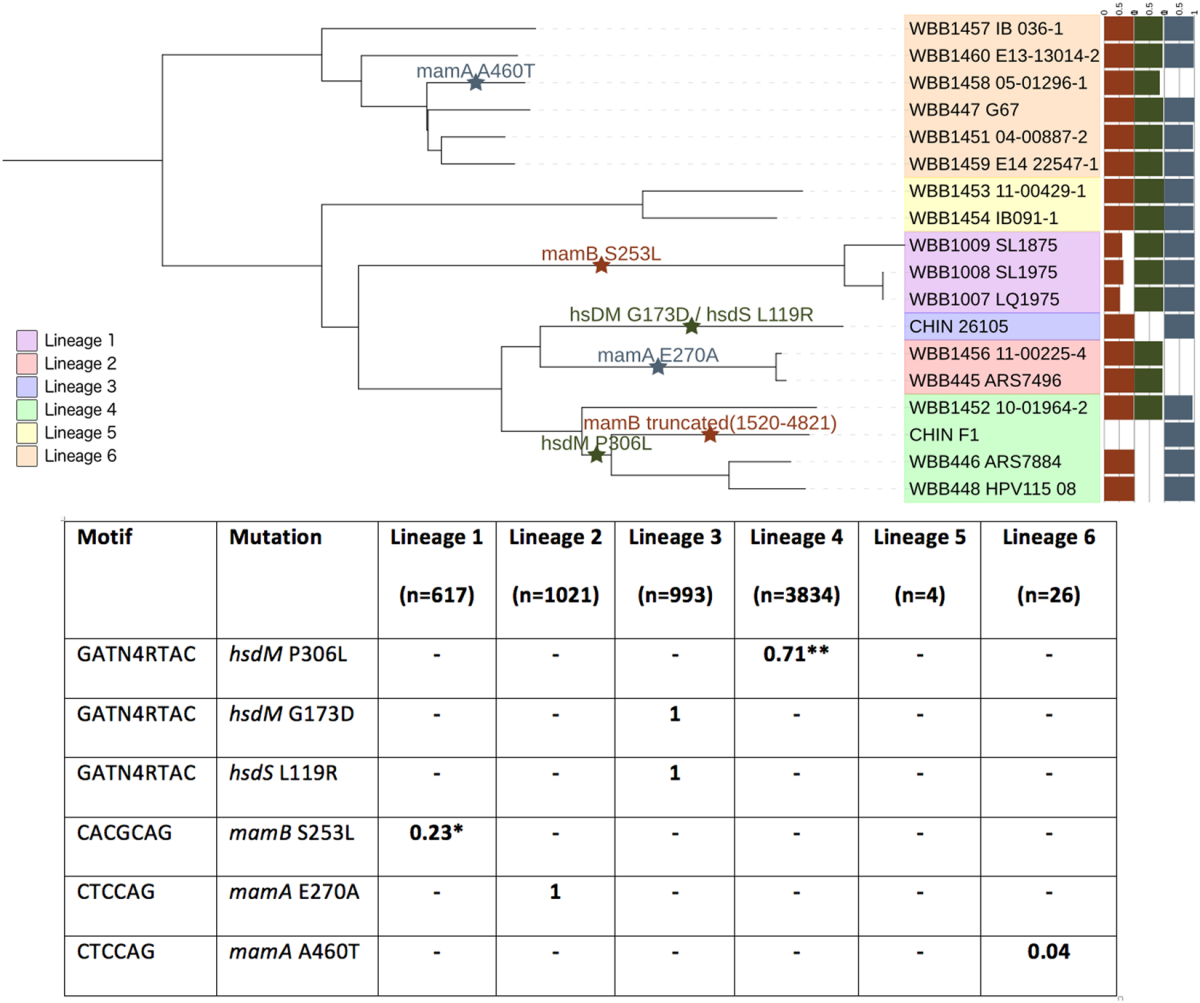
Phylogeny of the *Mycobacterium tuberculosis* complex isolate consensus sequences (n = 18) annotated with loss of function mutations in MTase genes. A maximum likelihood phylogenetic tree, with the % of methylated motifs and potential loss of function mutations in MTase genes annotated. Allele frequencies of putative methylation related mutations across a global collection of *M*. *tuberculosis* isolates; *EAI6 stains, **lineages 4.3 to 4.9, - indicates absence.

Sixteen samples representing the lineages 1, 2, 4, 5 and 6 were sequenced on the PacBio platform (Supplementary Table 1, n = 16), and supplemented by raw sequence data for a lineage 3 strain and a H37Rv strain (CHIN_F1) from earlier work (n = 2)^8^. High quality assemblies (no. contigs <10) were generated for the 18 isolates, with most isolates assembled into one contig (median n50 = 4.38 Mb, median genome length = 4.42 Mb). After aligning to the H37Rv reference, we found 10,353 unique small variant sites, with 50.7% of positions having alternate alleles in only one sample. A maximum likelihood tree was constructed using the variants (Fig. 1) and demonstrated the expected clustering by lineage, with two lineage 1 strains (WBB1008_SL1975, WBB1007_LQ1975) being near identical.

The error rate in the PacBio consensus sequences was assessed in three isolates (Supplementary Table 1; WBB446_ARS7884 (LAM strain, lineage 4), WBB448_HPV115_08 (LAM strain, lineage 4), WBB445_ARS7496 (Beijing strain, lineage 2)) that also had Illumina short read data with high coverage genome-wide (>50-fold). Alignment of the short reads to the consensus sequences revealed low numbers of discordant SNPs (range: 0–6), but slightly higher numbers of discordant insertions and deletions (indels) (range: 2 to 26) due to incorrect assembly at homopolymeric sites in the genome. More generally, further analysis of these isolates revealed the advantages of using lineage-specific reference genomes. First, using sets of 100 independent strains in each lineage^2^, there was a marginal improvement in the number of reads mapped compared to using an alignment to H37Rv (mean increase: lineage 1 0.47%, lineage 2 0.33%, lineage 3 0.25%). As *M*. *tuberculosis* has a very clonal genome, most of the genome shares near 100% identity across lineages, and therefore large improvements in overall mappability would not be expected. Second, we considered strain-specific regions in the highly variable *PE_PGRS3/4* and *PE_PGRS17/18* genes, which were hypothesised from *de novo* assembly analysis to have undergone a large genomic rearrangement in Beijing strains^10^. The PacBio consensus sequence confirmed the large re-arrangements in WBB445_ARS7496 (Beijing). These re-arrangements could be identified in coverage profiles through mapping WBB445_ARS7496 short reads to its own PacBio consensus sequence, but not to the H37Rv reference or other non-Beijing study consensus sequences (Supplementary Figure 1).

Annotation of the new reference genomes using prokka software^11^, guided by H37Rv protein sequences, revealed differences in the number of genes (range: 4028 to 4217). The CHIN_F1 strain (H37Rv) had a greater number of inferred genes (4217) than the H37Rv reference (ASM19595v2, 4093 genes), which may indicate that the automatic annotation software could be over-estimating numbers of genes. However, overall, there was a high degree of conservation among isolates across orthologous groups of genes (3666/4250, 86%). Hierarchical clustering of isolates using the number of shared orthogroups as a metric of genetic distance, revealed expected lineage-specific clustering, except for the CHIN_F1 strain which clustered outside lineage 4 and closer to lineage 3 (Supplementary Figure 2). To investigate differences in the gene content between sequenced strains, we performed pairwise alignments of the assemblies to the H37Rv reference, and scanned for regions of difference (with respect to the reference). We found 90 unique large deletions (minimum size of 1000 bp) at a gene-level (Supplementary Figure 3, Supplementary data 1). This set of novel deletions included 35 genes spanning from *mutA* to *Rv1523* in the lineage 5 strain (WBB1454_IB091-1). These large sequence polymorphisms could potentially explain phenotypic differences between strains and require further investigation.

### Methylation motif analysis

Using the Modification and Motif Analysis pipeline in the SMRT portal (https://github.com/PacificBiosciences/SMRT-Analysis), Pacbio sequence data can be used to robustly reveal methylation sites. A variable number of motifs (range: 3-13) were found per isolate, with 45 unique motifs discovered across the entire dataset of 18 isolates. Three high quality methylated motifs (quality value score >100) were detected across almost all isolates: CACGCAG (17/18 isolates), GATN4RTAC (14/18), and CTCCAG (15/18) (Table 1). Partner motifs for GATN4RTAC and CTCCAG were also found indicating methylation on both the forward and reverse strand, while CACGCAG is only hemi-methylated as no partner motif was found. These motifs have previously been reported^8,12^. The number of occurrences of each motif was found to vary slightly across isolates (range: GATN4RTAC 349-366, CACGCAG 811-828, CTCCAG 1928-1957).

**Table 1:** Methylation of motifs and their proportion in the genome sequence assemblies of each isolate.

Isolate [lineage]		CACGCAG	GATNNNNRTAC	GTAYNNNNATC	CTCCAG	CTGGAG
	WBB1457_IB_036-1 [6]	793/811 (0.98)	332/351 (0.95)	332/351 (0.95)	1885/1934 (0.97)	1828/1934 (0.95)
	WBB1460_E13-13014-2 [6]	799/813 (0.98)	328/350 (0.94)	327/350 (0.93)	1892/1937 (0.98)	1825/1937 (0.94)
	WBB1458_05-01296-1 [6]	799/813 (0.98)	294/349 (0.84)	290/349 (0.83)	0/1932 (0.00)	0/1932 (0.00)
	WBB447_G67 [6]	814/814 (1.00)	338/352 (0.96)	336/352 (0.95)	1923/1933 (0.99)	1922/1933 (0.99)
	WBB1451_04-00887-2 [6]	802/812 (0.99)	328/349 (0.94)	325/349 (0.93)	1842/1933 (0.95)	1801/1933 (0.93)
	WBB1459_E14_22547-1 [6]	802/811 (0.99)	328/349 (0.94)	329/349 (0.94)	1891/1934 (0.98)	1833/1934 (0.95)
	WBB1453_11-00429-1 [5]	814/828 (0.98)	357/362 (0.99)	355/362 (0.98)	1889/1942 (0.97)	1825/1942 (0.94)
	WBB1454_IB091-1 [5]	807/823 (0.98)	356/358 (0.99)	353/358 (0.99)	1874/1929 (0.97)	1819/1929 (0.94)
	WBB1009_SL1875 [1]	492/826 (0.60)	345/360 (0.96)	341/360 (0.95)	1942/1957 (0.99)	1885/1957 (0.96)
	WBB1008_SL1975 [1]	526/826 (0.64)	344/360 (0.96)	345/360 (0.96)	1945/1956 (0.99)	1906/1956 (0.97)
	WBB1007_LQ1975 [1]	434/826 (0.53)	345/360 (0.96)	338/360 (0.94)	1943/1956 (0.99)	1893/1956 (0.97)
	CHIN_26105 [3]	823/824 (1.00)	0/362 (0.00)	0/362 (0.00)	1939/1954 (0.99)	1942/1954 (0.99)
	WBB1456_11-00225-4 [2]	813/826 (0.98)	344/366 (0.94)	349/366 (0.95)	0/1949 (0.00)	0/1949 (0.00)
	WBB445_ARS7496 [2]	824/824 (1.00)	339/363 (0.93)	340/363 (0.94)	0/1947 (0.00)	0/1947 (0.00)
	WBB1452_10-01964-2 [4]	798/817 (0.98)	332/358 (0.93)	321/358 (0.90)	1828/1947 (0.94)	1748/1947 (0.90)
	CHIN_F1 [4]	0/820 (0.00)	0/361 (0.00)	0/361 (0.00)	1937/1948 (0.99)	1937/1948 (0.99)
	WBB446_ARS7884 [4]	817/817 (1.00)	0/357 (0.00)	0/357 (0.00)	1932/1933 (1.00)	1927/1933 (1.00)
	WBB448_HPV115_08 [4]	814/814 (1.00)	0/355 (0.00)	0/355 (0.00)	1927/1928 (1.00)	1924/1928 (1.00)

The phylogenetic relationship and fraction of motifs methylated for each strain. Most values are close to either 0.95 or 0 indicating the presence or complete absence of methylation, however, all lineage 1 strains had approximately half of their CACGCAG motif methylate.

By considering the motifs across all isolate genome assemblies and inspection of the raw inter-pulse duration (IPD) ratios at each nucleotide position in the motif, we found that isolates where the motif was present but had no evidence of modification across nucleotides (Supplementary Figure 4). There was some variability across and within strain types in the percent of motifs methylated. In particular, although motifs were mostly close to 100% (or alternatively 0%) methylated, three isolates had a substantially different percentage for the CACGCAG motif (median (range) %: 60.0 (52.5–63.7)) (Fig. 1). Methylation of the other two motifs (GATN4RTAC, CACGCAG) did not seem affected in these isolates (range 93.9–99.3%).

To explain the differences in methylation pattern we identified mutations in methyltransferase genes that have been associated with each motif (GATN4RTAC: *hsdS*.*1*, *hsdM* and *hsdS;* CTCCAG: *mamA*; CACGCAG: *mamB*)^8^ (Fig. 1). In particular, we scanned for mutations that were present in methylation-deficient isolates, as identified through analysis of PacBio data, which could putatively explain loss of function in the respective methyltransferase. For the GATN4RTAC motif we found three unique mutations in four isolates with an absence of methylation, confirming those identified in previous reports^8^. Three methylation-absent isolates had the presence of the *hsdM* P306L mutation. Additionally, one sample had two mutations which were not present in any other isolates: *hsdM* G173D and *hsdS* L119R. Three samples did not exhibit any methylation at the CTCCAG motif, and we identified three unique mutations in *mamA*, one of which was present in two samples. One isolate had an E270A mutation and frameshift deletion at position 1257, however through phylogenetic ancestral reconstruction we deduced that the E270A mutation occurred before the deletion (Fig. 1). The two other isolates had E270A and previously uncharacterised A460T mutations, respectively. For the CACGCAG motif, the CHIN_F1 strain has a truncated *mamB* gene which has been reported elsewhere^8^, and verified here. Additionally, we found all three lineage 1 strains, which exhibited ~50–60% methylation, to have a novel S253L mutation in *mamB*.

### Pathway analysis

To look for the non-random association of methylation sites and protein families or biological pathways we performed a pathway analysis using DAVID software^13^. Each of the three motifs was considered individually. Motifs were associated with genes based on overlap with the coding region. For GATN4RTAC, we found an enrichment of cell membrane associated genes (Bonferroni corrected P-value (*P) = 0.021) and plasma associated genes (*P = 0.023) in motif-containing genes compared to genes without the motifs. For CTCCAG, motif-containing genes were enriched for nucleotide binding (*P = 2.85e-14) and cell wall (*P = 1.90e-5) among others (Supplementary Table 2). For the CACGCAG motif we found several enriched pathways involved in fatty acid and polyketide synthesis (*P = 9.26E-05) among others. DAVID software was used to test whether there was targeted absence of methylation of genes in a specific pathway. Genes with an absence of methylation in excess of 60% of the isolates were compared against all *M*. *tuberculosis* genes to look for enrichment of specific pathways. This analysis was performed on an overall and per-lineage basis. No pathways reported significant results (*P > 0.05). When comparing motif-containing unmethylated to motif-containing methylated genes on a lineage basis we did not find any significantly enriched pathways, although the small number of isolates is likely to lead to reduced power to detect true enriched pathways. To assess differential methylation of promoter sequences we looked for overlap of the motifs to the reported −10 regions of transcriptional start sites^14^. Only the CTGGAG motif was found to overlap with promoter sequences in eight genes (*Rv0102*, *Rv0142*, *Rv0898c*, *Rv1049*, *corA*, *Rv3083*, *whiB7 and lipV*). These promoter sites were all methylated for isolates with active *mamA*, except for *corA* in WBB1451_04–00887–2, which was deleted. A pathway analysis of these genes revealed no common function.

### Motifs in a global context

**Figure 2:**
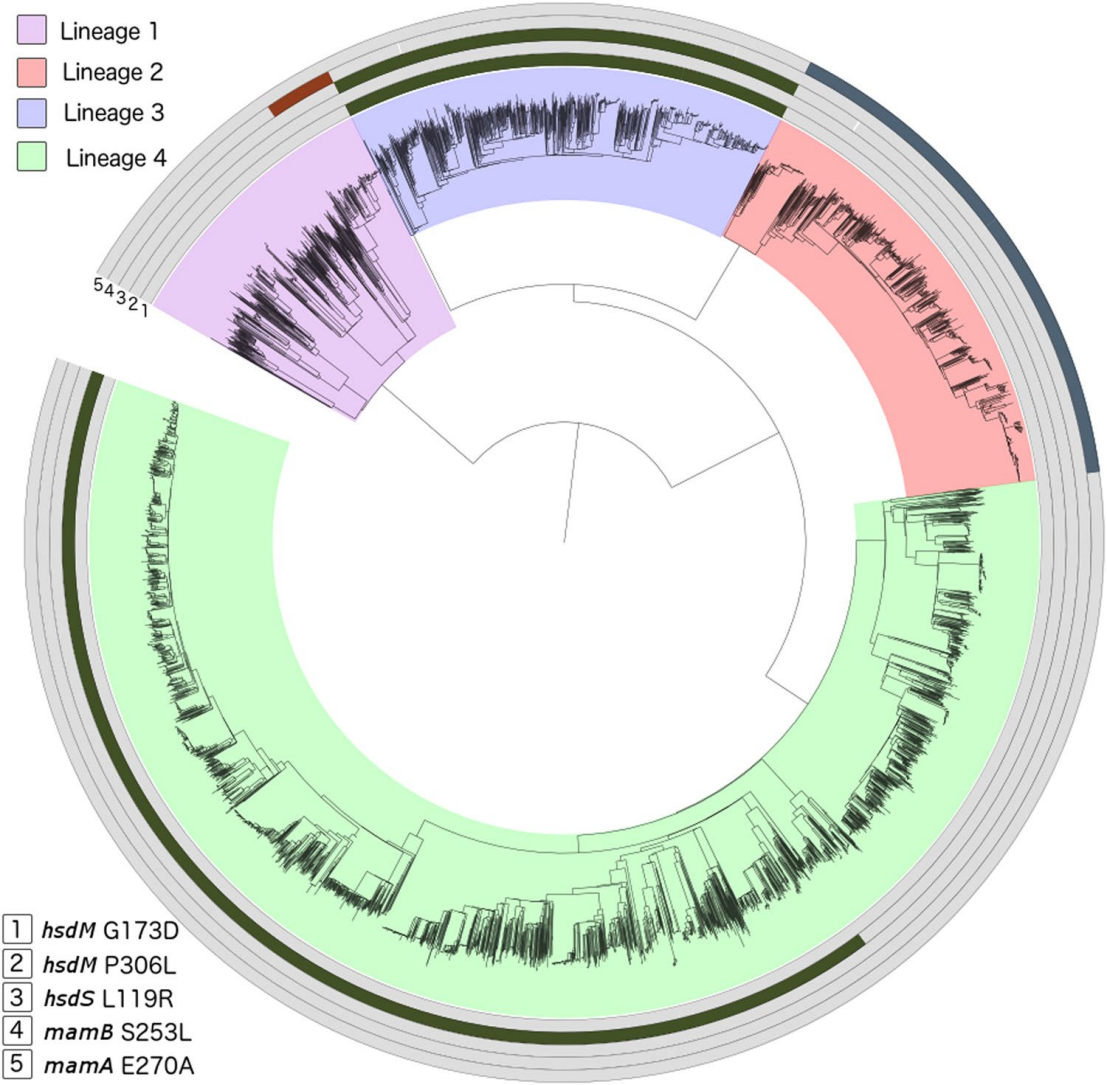
Five methylation-affecting mutations in a global collection of isolates (n = 6465; lineage 1 617 (9.5%), lineage 2 1021 (15.8%), lineage 3 993 (15.4%), lineage 4 3834 (59.3%)^20^).

To describe the six mutations we identified as affecting methylation in a global context, we analysed a large collection (n = 6,465) of isolates representing lineages 1 (9.5%), 2 (15.8%), 3 (15.4%) and 4 (59.3%). We also analysed lineage 5 (n = 4) strains and lineage 6 (n = 26) strains, a combination of our own data and those described elsewhere^15^. We found five of the six mutations identified above in the global dataset, occurring predominantly in single lineages with low frequencies in other lineages (Fig. 1), and originating at unique positions in the phylogeny (Fig. 2). None of the six mutations were found in the lineage 5/6 dataset, except for the isolate in which we originally found the *mamA* 460 T mutation. The *mamA* A460T is likely to be specific to a subclade of lineage 6. Three mutations affecting the GATN4RTAC motif were found at high allele frequency (*hsdM* G173D: 0.15, *hsdM* P306L: 0.42, *hsdS* L119R: 0.15) and affected ~57% of the isolates. The *hsdM* P306L mutation is a phylogenetically deep mutation which occurs in a sub-clade of lineages 4.3 to 4.9 (H3, H4, LAM, LAM1, LAM10-CAM, LAM11-ZWE, LAM3, LAM4, LAM9, S, T1, T2, T2-Uganda, T3, T4, T5). The *hsdM* G173D and *hsdS* L119R mutations are present in all lineage 3 isolates. The *mamB* S253L mutation affecting the CACGCAG motif is present only in a subclade of lineage 1 (EAI6). The *mamA* E270A mutation affecting the CTCCAG motif is present in all lineage 2 strains. Assuming that these mutations do indeed cause the absence of methylation on the genome there is a stark difference between the motifs in the lineages and number of samples which have active methylation.

## Discussion

We have presented 16 new reference genomes and methylomes of strains with diverse genetic backgrounds. The ability of PacBio technology to produce long reads leads to complete genome assemblies that capture both small and large genomic variations and have a very high accuracy at repetitive regions such as the *PE/PPE* genes. Most whole genome sequencing projects have focused on lineages 1 to 4 because of their prevalence and global distribution, however recent studies have shown a large amount of genetic diversity to be present within lineages 5 and 6^15^. Additionally, an intriguing question remains why lineages 5 and 6 are localised to West Africa and have not spread globally. The lineage specific variants and differences in gene content (including the *PE/PPE* genes) reported here, building on previous work^3^, could potentially play a role in specific host population adaptation. We present, to our knowledge, the first complete lineage 5 reference genomes, and increase substantially the number of lineage 6 reference genomes available. These references will be useful in future whole genome sequencing projects that investigate the genetic diversity of lineage 5 and 6 strains, as well as strain-host genetic interactions. By aligning Illumina reads to our references we find there to be a small increase in the number of reads mapping (0.25–0.47%), particularly in genomic regions where sequences are either not present or highly variable in the H37Rv reference. By performing automatic annotation and clustering of protein sequence into clusters of orthologues, we report a significant difference in the gene content between strains. Overall, these new reference sequences could serve to improve the accuracy of resequencing experiments by facilitating lineage-specific mapping at highly variable regions and to improve our understanding of large structural variations such as novel insertions, as well as rearrangements between lineages.

The PacBio technology allowed us to characterise the methylation at sites along the genome. Across the 18 isolates, three motifs are methylated to varying degrees. While most isolates had close to 100% methylation with an active MTase, the three lineage 1 isolates had 53–64% methylation at the CACGCAG motif while maintaining near 100% methylation on both other motifs. Another study using PacBio technology reported the identification of five mutations that associate with the absence of methylation^8^. Using an independent set of samples we have replicated these results and additionally identified the *mamA* A460T and *mamB* S253L mutations, potentially involved in disrupting methylation; although these findings require further validation. Most importantly, we analysed these mutations in the context of a large global dataset of strains and report on the lineage specificity of methylation. Five of these mutations were present in a large global phylogeny consisting of *M*. *tuberculosis* lineages (1–4) strains. The frequency of the potential loss of function mutations is reasonably high. For example, the three mutations (*hsdM* G173D, *hsdM* P306L and *hsdS* L119R) affecting the GATN4RTAC motif methylation were present in all available lineage 3 (all sub-lineages) strains, as well as across a larger number of lineage 4 sub-lineages (including H3, H4, LAM, LAM1, LAM10-CAM, LAM11-ZWE, LAM3, LAM4, LAM9, S, T1, T2, T2-Uganda, T3, T4, T5), but absent in other lineages. Similarly, the other motifs (CTCCAG and CACGCAG) have a lower frequency of loss of function mutations, but are also strain specific. Follow-up investigation is required to provide an insight into the essential and functional nature of methylation, and its association with the different motifs. Interestingly all lineage 2 strains, which have been reported to be highly virulent^16^, lack methylation in the most abundant motif (CTCCAG) putatively due to the *mamA* E270A mutation. Differential methylation patterns could provide a possible explanation for the increased virulence in this clade, as genetic distance is relatively small. Interestingly the *dosR* regulon has been shown to be upregulated in modern Beijing strains^17^. Both the *dosR* regulon and the methylation of the *mamA* motif may be involved in hypoxia^7,17^. Whether their co-occurrence is related has yet to be investigated. Similarly, the *mamB* S253L mutation related to the CACGCAG motif seems only present in EAI6 strains, and whilst little is known whether these strains are more virulent than other lineage 1 “ancient” strains, they have spread globally and have been associated with recent outbreaks^18^, unlike other lineage 1 strains.

It has been hypothesised that DNA methylation influences transcription^9^ and therefore it would be expected to see a differences in transcriptional profiles of genes where there is differential methylation. Additionally, although no correlation was found with drug resistance (data not shown), transcriptional regulation by DNA methylation could potentially contribute towards observed strain-specific differences in the acquisition of mutations involved in drug resistance^19^. Whilst, our work has shed new light on *M*. *tuberculosis* methylation, future work should consider more diverse strains and integrate transcriptomic data to further elucidate underlying biological mechanisms and associating them with virulence and other important phenotypic outcomes including antibiotic resistance.

## Materials and Methods

### Samples and SMRT sequencing

All live *M*. *tuberculosis* isolates were handled in containment level 3 facilities in the collaborating centres. DNA was extracted from *M*. *tuberculosis* cultures of clinical samples, processed using methods described elsewhere^2,3,20^. These methods have been demonstrated to kill all bacteria. DNA was then confirmed as bacteria free, before sending to the Genome Institute of Singapore for sequencing. Samples were sequenced using Pacific Biosciences (PacBio) RSII long read technology. Additionally, raw data for two isolates was downloaded from the SRA project SRP064893 to be included in the current study. All raw sequencing data are available, and the study accession numbers are listed in Supplementary Table 1.

### Bioinformatic analysis

Sequencing reads were assembled using Hierarchical Genome Assembly Process HGAP2 implemented in the SMRT Portal software suite. Short low confidence contigs (length <1000 or identity <90%) were removed from subsequent analyses. Overlap between the start and end of large contigs were found by self-aligning using *Mummer* software (mummer.sourceforge.net) and removed using in-house scripts. Contigs were aligned, scaffolds inferred, reordered and, if needed, reverse-complemented according to the H37Rv reference using the *mummer* tool and in-house scripts. Following this the reads were realigned to the scaffolds to improve the consensus concordance. The final consensus genome for each sample was annotated using *prokka* automatic annotation tool^11^ using the H37Rv protein sequences to annotate the genes found. *Mummer* ^21^ software was used to align the consensus against H37Rv to identify small variants (SNPs and indels). *Mauve software* ^22^ was used to perform pairwise alignments to identify large deletions. Methylation analysis was performed using the Modification and Motif Analysis pipeline in SMRT portal, and outputted motifs of interest. All high-quality motifs were used in further downstream analysis. A maximum likelihood phylogenetic tree was built using *RAxML* ^23^ with all polymorphic SNP sites found. Pathway analysis was performed by assigning a gene to each motif found in a genome. Genes were assigned using overlap with the coding region or promoter of a gene. Statistical enrichment analysis was performed using *DAVID* software^14^ and compared: (i) all motif-containing genes to all *M*. *tuberculosis* genes; (ii) all un-methylated genes to all motif-containing genes. To identify mutations within lineages 5 and 6, genome assemblies were downloaded from *genbank* ^15^ and aligned to the H37Rv reference using the *mummer* tool with default parameters. Variants were then called using the *snp-snps* algorithm, with the “-C” parameter invoked, leading to the reporting of variants from unambiguous alignments.

## Electronic supplementary material

Supplementary information

Supplementary Dataset 1
