# Supplementary material for: Methylation in Mycobacterium tuberculosis is lineage specific with associated mutations present globally
Source: Sci Rep. 2018 Jan 9;8:160. doi: 10.1038/s41598-017-18188-y (PMC5760664; doi:10.1038/s41598-017-18188-y)
Supplement: Supplementary file 1 — Supplementary information [file 41598_2017_18188_MOESM1_ESM.doc]

**Methylation in *Mycobacterium tuberculosis* is lineage specific with associated mutations present globally**

Jody Phelan^1^, Paola Florez de Sessions^2,*^, Leopold Tientcheu^3,*^, Joao Perdigao ^4,*^, Diana Machado^,5*^, Rumina Hasan^6^, Zahra Hasan^6^, Indra L. Bergval ^7^, Richard Anthony ^7^, Ruth McNerney ^1,8^, Martin Antonio ^3^, Isabel Portugal ^4^, Miguel Viveiros ^5^, Susana Campino^1^, Martin L. Hibberd^1,2,**^, Taane G Clark^1,9,**^

^1^ Faculty of Infectious and Tropical Diseases, London School of Hygiene and Tropical Medicine, London, United Kingdom

^2^ Genome Institute of Singapore, Biopolis, Singapore

^3^ Vaccines and Immunity Theme, Medical Research Council Unit, Fajara, The Gambia

^4^ iMed.ULisboa - Research Institute for Medicines, Faculdade de Farmácia, Universidade de Lisboa, Lisboa, Portugal

^5^ Unidade de Microbiologia Médica, Global Health and Tropical Medicine, Instituto de Higiene e Medicina Tropical, Universidade Nova de Lisboa, Lisboa, Portugal

^6^ Department of Pathology and Laboratory Medicine, The Aga Khan University, Karachi, Pakistan

^7^ Royal Tropical Institute, KIT Biomedical Research, Amsterdam, The Netherlands

^8^ Lung Infection and Immunity Unit, UCT Lung Institute, University of Cape Town, Cape Town, South Africa.

^9^ Faculty of Epidemiology and Population Health, London School of Hygiene and Tropical Medicine, London, United Kingdom

* joint authors

** joint corresponding authors

**Supplementary table 1**

The isolates analysed

| Isolate ID | Country | Source | N50 | Num. Contigs | Genome length | Lineage | Sub-lineage | SRA accession |
| --- | --- | --- | --- | --- | --- | --- | --- | --- |
| WBB1007_LQ1975 | Mozambique | Sequenced | 4450176 | 1 | 4450176 | 1 | 1.1.3 (EAI6) | PRJEB21888 |
| WBB1008_SL1975 | Mozambique | Sequenced | 4467776 | 1 | 4467776 | 1 | 1.1.3 (EAI6) | PRJEB21888 |
| WBB1009_SL1875 | Mozambique | Sequenced | 4438486 | 1 | 4438486 | 1 | 1.1.3 (EAI6) | PRJEB21888 |
| WBB1456_11-00225-4 | Gambia | Sequenced | 4415343 | 1 | 4415343 | 2 | 2.2.1 (Beijing)* | PRJEB21888 |
| **WBB445_ARS7496** | **Portugal** | **Sequenced** | **4415871** | **3** | **4446789** | **2** | **2.2.1 (Beijing)*** | PRJEB21888 |
| CHIN_26105 | China | SRA | 4440106 | 1 | 4440106 | 3 | 3 (CAS)* | SRP064893 |
| WBB1452_10-01964-2 | Gambia | Sequenced | 4416076 | 2 | 4430073 | 4 | 4.1.2.1 (Haarlem)* | PRJEB21888 |
| **WBB446_ARS7884** | **Portugal** | **Sequenced** | **4375931** | **3** | **4396369** | **4** | **4.3.4.2 (LAM)*** | PRJEB21888 |
| **WBB448_HPV115_08** | **Portugal** | **Sequenced** | **4385381** | **1** | **4385381** | **4** | **4.3.4.2 (LAM)*** | PRJEB21888 |
| CHIN_F1 | China | SRA | 4125500 | 5 | 4438875 | 4 | 4.9 (T1-H37Rv) | SRP064893 |
| WBB1453_11-00429-1 | Gambia | Sequenced | 4430643 | 1 | 4430643 | 5 | 5 (Afr2/3) | PRJEB21888 |
| WBB1454_IB091-1 | Nigeria | Sequenced | 3865667 | 3 | 4419358 | 5 | 5 (Afr2/3) | PRJEB21888 |
| WBB1451_04-00887-2 | Gambia | Sequenced | 716074 | 6 | 4393399 | 6 | 6 (Afr1) | PRJEB21888 |
| WBB1457_IB_036-1 | Nigeria | Sequenced | 2521417 | 4 | 4387174 | 6 | 6 (Afr1) | PRJEB21888 |
| WBB1458_05-01296-1 | Gambia | Sequenced | 2446180 | 2 | 4369685 | 6 | 6 (Afr1) | PRJEB21888 |
| WBB1459_E14_22547-1 | Gambia | Sequenced | 4382305 | 2 | 4384418 | 6 | 6 (Afr1) | PRJEB21888 |
| WBB1460_E13-13014-2 | Gambia | Sequenced | 2963146 | 4 | 4413823 | 6 | 6 (Afr1) | PRJEB21888 |
| WBB447_G67 | Guinea-Bissau | Sequenced | 2330737 | 3 | 4388314 | 6 | 6 (Afr1) | PRJEB21888 |

**Bolded** isolates that also have Illumina short read data; Sub-lineages inferred using barcoding SNPs^3^; Afr = *M. africanum;* * known to be highly virulent^5^; SRA short read archive

**Supplementary table 2**

Pathway analysis of genes containing motifs

| **Motif** | **Ontological annotation** | | **Count** | **% of genes in pathway** | **Fold Enrichment** | **P-value*** |
| --- | --- | --- | --- | --- | --- | --- |
| CTCCAG | ATP-binding |  | 178 | 12.4 | 1.6 | 2.85E-14 |
| CTCCAG | Cell wall |  | 327 | 22.8 | 1.2 | 1.90E-05 |
| CTCCAG | Plasma membrane | | 632 | 44 | 1.1 | 1.13E-04 |
| CTCCAG | Phosphoprotein | | 52 | 3.6 | 1.7 | 9.23E-04 |
| CTCCAG | P-loop containing nucleoside triphosphate hydrolase | | 107 | 7.5 | 1.5 | 0.001 |
| CTCCAG | Intracellular |  | 45 | 3.1 | 1.7 | 0.001 |
| CTCCAG | Transferase |  | 237 | 16.5 | 1.2 | 0.001 |
| CTCCAG | Carbon metabolism | | 69 | 4.8 | 1.4 | 0.005 |
| CTCCAG | Cytoplasm |  | 151 | 10.5 | 1.3 | 0.007 |
| CTCCAG | Cytosol |  | 239 | 16.6 | 1.2 | 0.008 |
| CTCCAG | Glyoxylate & dicarboxylate metabolism | | 30 | 2.1 | 1.7 | 0.015 |
| CTCCAG | Fatty acid / polyketide synthesis | | 21 | 1.5 | 2.1 | 0.017 |
| CTCCAG | Ligase |  | 65 | 4.5 | 1.5 | 0.024 |
| CACGCAG | Fatty acid / polyketide synthesis | | 18 | 2.5 | 3.5 | 9.26E-05 |
| CACGCAG | Nucleotide-binding | | 90 | 12.5 | 1.4 | 0.02 |
| CACGCAG | Cytosol |  | 128 | 17.7 | 1.3 | 0.048 |
| GATNNNNRTAC | Cell membrane | | 59 | 17.9 | 1.6 | 0.021 |
| GATNNNNRTAC | Plasma membrane | | 149 | 45.3 | 1.2 | 0.023 |

Motifs were assigned to genes by finding overlap with coding regions. If found in intergenic regions the motif was assigned to the gene with the closest promoter. Genes at which the motif was found in >60% of the isolates were used to look for enrichment of pathways; * Bonferroni corrected P-value (P* in main text).

**Supplementary Figure 1**

Differences in coverage in the *PE_PGRS3/4* and *PE_PGRS17/18* highly variable regions when comparing mapping of the WBB445_ARS7496 Illumina reads to the H37Rv reference and WBB445_ARS7496 Beijing reference described in this publication. The genes on the H37Rv reference used can be seen on the bottom track. The GC content and the uniqueness (1 = unique, < 1 non-unique) of a region can influence the coverage across the region and are plotted on the middle panels. The coverage is plotted on the top panel. The H37Rv mapping results are plotted on the left, while the WBB445_ARS7496 assembly results are plotted on the right.

1. ***PE_PGRS3* region**

**
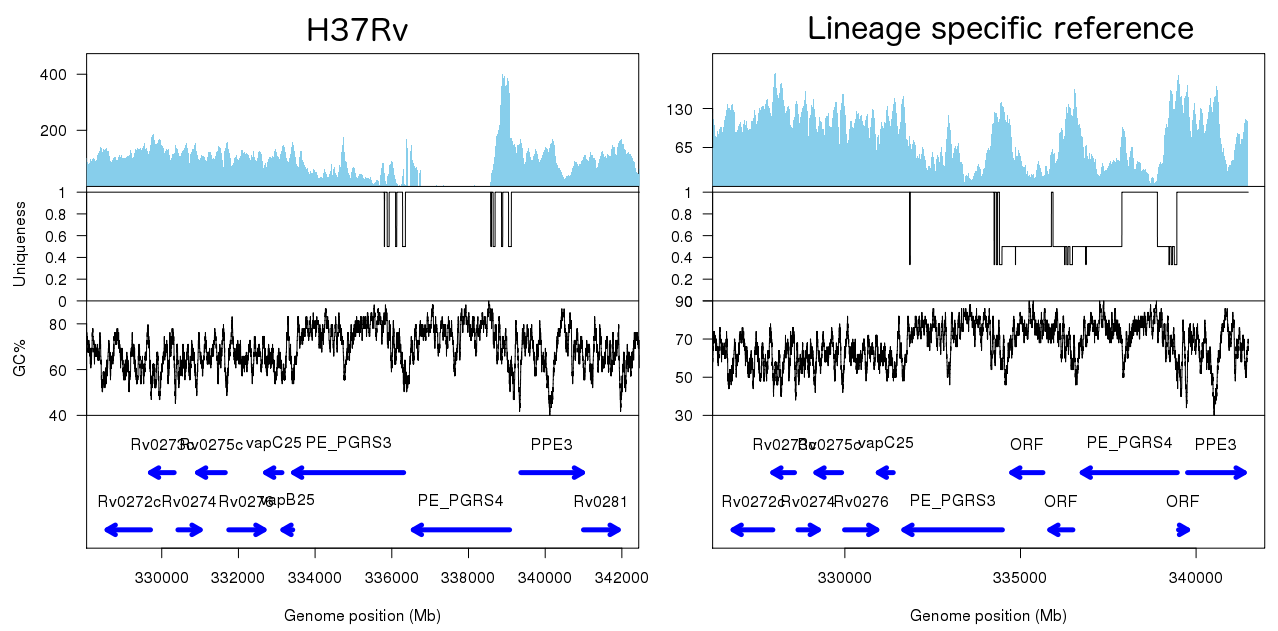
**

Higher coverage is seen across both the *PE_PGRS3* and *PE_PGRS4* when mapping to the new lineage specific reference. Additionally, two new open reading frames have been introduced between the two genes.

**B) *PE_PGRS17/18* region**

**
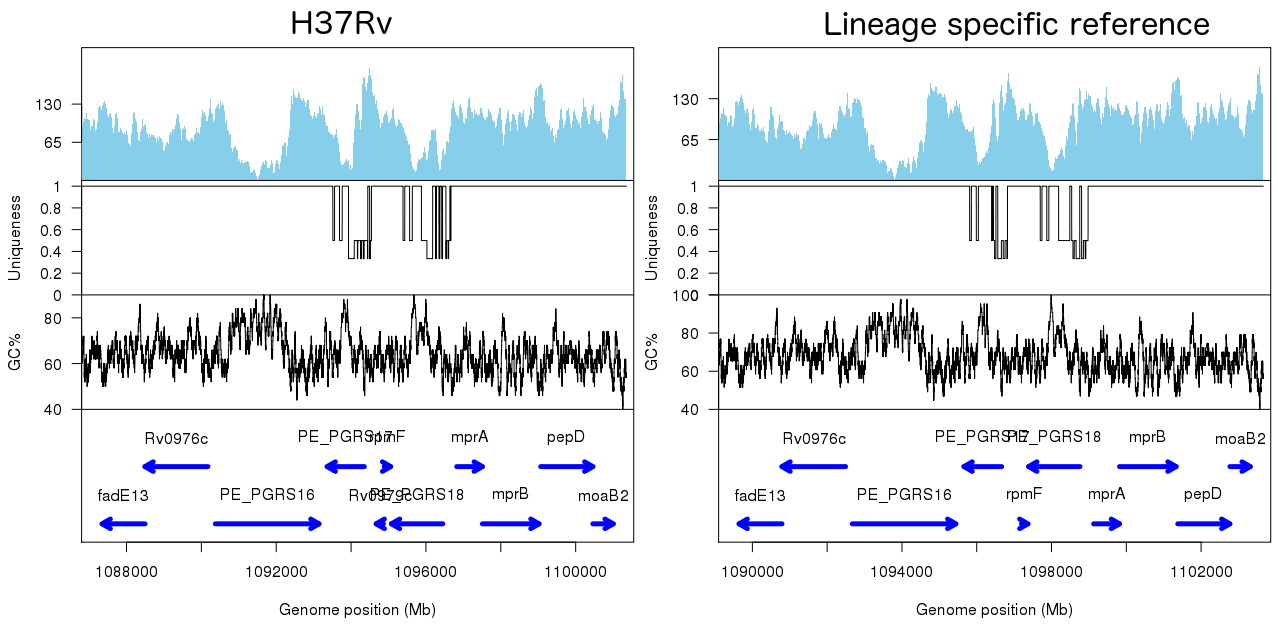
**

Only slight changes in genomic coverage were detected, indicating that the lack of coverage across these genes is mostly due to the high GC content in some regions coupled with the fact that some regions are non-unique.

**Supplementary figure 2**

**Hierarchical clustering and heat map visualisation of shared number of orthogroups (groups of orthologous proteins).**

Correct clustering can be observed for all isolates except CHIN_F1 (H37Rv strain) which is located outside lineage 4 and closer to lineage 3. **
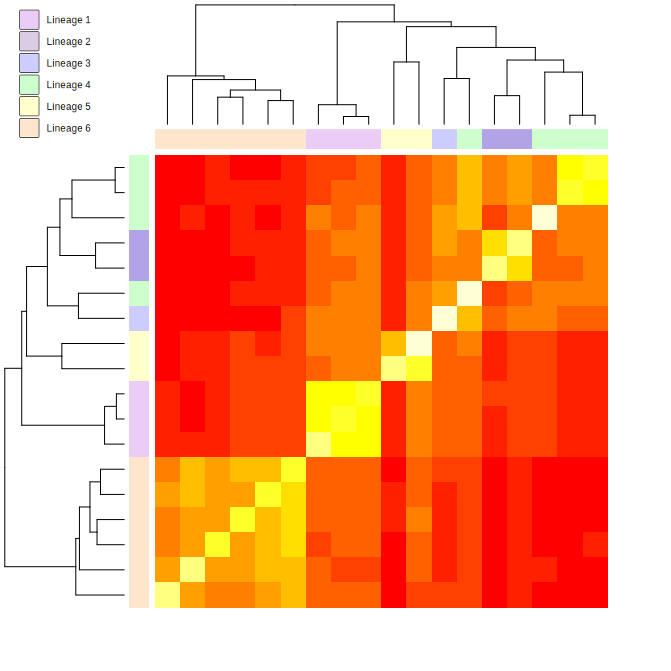
**

**Supplementary figure 3**

**A visualisation of all the large deletions (as compared to the reference, see Supplementary data 1 for a list).**

**
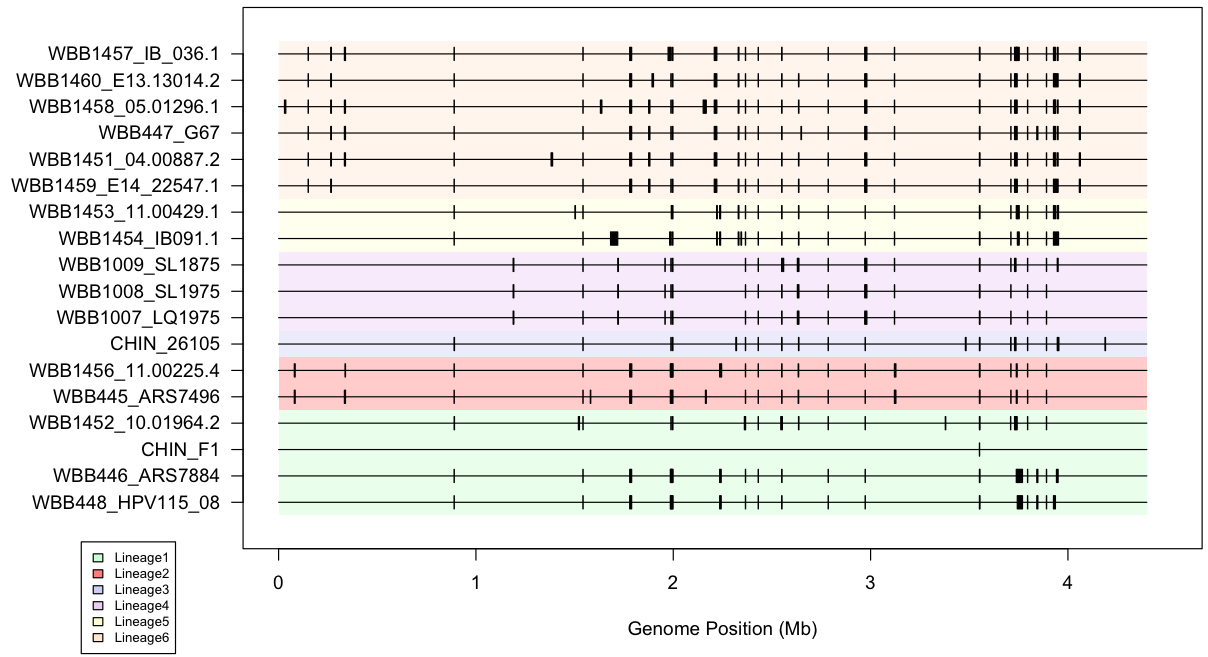
**

**Supplementary figure 4**

Inter-pulse duration (IPD) ratios across motifs in unmethylated isolates (left column) and methylated isolates (right column): A) GATN4RTAC, B) CACGCAG and C) CTCCAG **
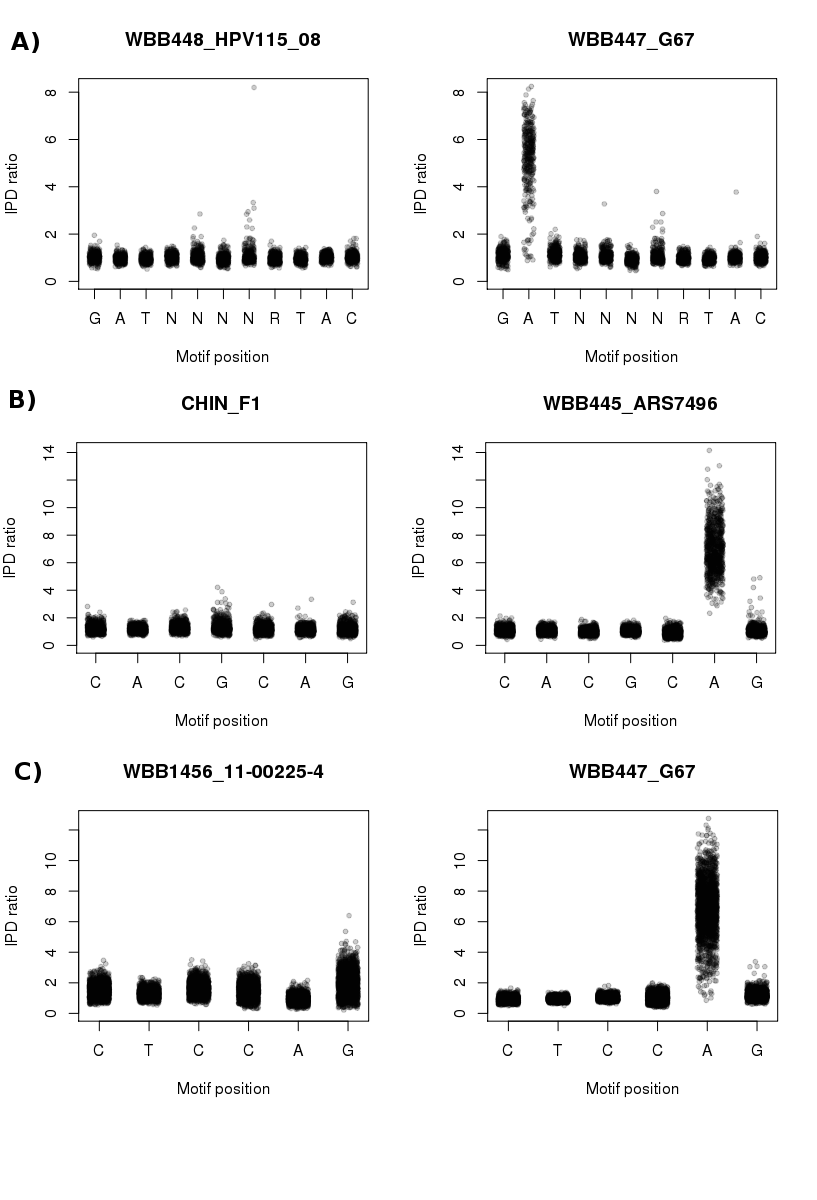
**
